# The Pathogenomics of the Respiratory *Mycoplasma bovis* Strains Circulating in Cattle Around the Texas Panhandle, USA

**DOI:** 10.3390/pathogens14020167

**Published:** 2025-02-08

**Authors:** Ethan P. Dudley, Matthew A. Scott, Hatem Kittana, Alexis C. Thompson, Robert Valeris-Chacin

**Affiliations:** 1 College of Veterinary Medicine and Biomedical Sciences, Texas A&M University, Canyon, TX 79015, USA; edudley01@exchange.tamu.edu (E.P.D.); mascott@cvm.tamu.edu (M.A.S.); 2 College of Veterinary Medicine, Kansas State University, Manhattan, KS 66506, USA; hkittana@vet.k-state.edu; 3 Texas A&M Veterinary Medical Diagnostic Laboratory, Canyon, TX 79015, USA; alexis.thompson@tvmdl.tamu.edu

**Keywords:** *Mycoplasma bovis*, virulence genes, Texas, long reads

## Abstract

Bovine respiratory disease (BRD) is a major economic and animal welfare issue in the beef industry. *Mycoplasma bovis* is one of the main causal organisms, particularly in chronic cases. Due to the difficulty of isolating *M. bovis* from clinical isolates, there is a lack of information on the genetic diversity of this pathogen in the Texas panhandle region of the United States. Therefore, our objective was to provide genome-level characterization of *M. bovis* isolated from the lung lesions of beef and dairy cattle in the Texas panhandle. Fifty-four isolates displaying mycoplasma-like growth were recovered from bovine lung lesions by the Texas Veterinary Medical Diagnostic Laboratory in 2021 and 2022. Of these isolates, 32 were determined to be *M. bovis* via species-specific qPCR using the *uvrC* gene. Long-read whole-genome sequencing was used to identify key virulence factors, antimicrobial resistance genes, and to assess the genetic diversity of these isolates. Fisher’s exact tests were used to identify associations between isolate characteristics and host metadata, including the state of origin, type of operation, animal age, and animal sex. Our results indicate that there is considerable genetic diversity among the *M. bovis* isolates, despite their shared geography in the Texas panhandle, though significant clustering based on host metadata was observed. Analysis of the pangenome showed that the *M. bovis* isolates in this study also harbor a diverse array of virulence genes, but no antimicrobial resistance genes were identified in this study.

## 1. Introduction

Bovine respiratory disease (BRD) is a significant economic and animal welfare burden on beef production in the United States. Bovine respiratory disease is a complex, multifactorial disease in which commensal bacteria in the respiratory tract proliferate and cause disease when stressors such as weaning, shipping, and viral co-infection weaken the host immune system [1,2]. In addition to death loss, BRD incurs several other production costs such as reduced weight gain, reduced feed efficiency, and the costs of treatment, vaccination, and antimicrobial metaphylaxis [3].

*Mycoplasma bovis* is a member of the class Mollicutes, which is characterized by the lack of a cell wall, a small genome size, and complex requirements for in vitro growth [4,5,6]. Historically, *M. bovis* has been associated with chronic cases of BRD characterized by caseonecrotic lung lesions and associated bronchopneumonia [7,8]. In addition to its role in BRD, *M. bovis* infections have also been associated with several other clinical manifestations, such as mastitis, arthritis, otitis, keratoconjunctivitis, meningitis, and endocarditis [6,9,10,11,12,13]. In recent years, *Mycoplasma bovis* has also been recognized as an emerging pathogen in North American bison [14]. Previous work has shown that *M. bovis* strains isolated from infected bison possess unique multi-locus sequence types compared to the isolates causing disease in cattle [15]. Unlike *M. bovis* isolated from cattle, which often acts in tandem with other pathogens to cause disease [16], *M. bovis* appears to act as a primary pathogen in bison, with greater virulence than is observed in healthy cattle experimentally challenged with *M. bovis* isolated from bison [14].

*Mycoplasma bovis* has several virulence factors associated with its adhesion to respiratory epithelial cells and evasion of the host immune system, such as variable surface proteins, adhesins, nucleases, biofilm formation, and hydrogen peroxide production [17,18,19]. Previous work has identified a number of genes that code for virulence-associated products, such as *milA*, *nox*, *nfo*, *metk*, and *dnaJ,* among others, many of which have multifunctional roles as both biosynthetic enzymes and virulence factors [20,21,22]. In addition to these virulence factors, the *M. bovis* genome contains a wide array of genes that code for variable surface lipoproteins (Vsps), which are highly immunogenic and capable of undergoing high-frequency phase variation, contributing to the ability of *M. bovis* to evade the host immune response [23,24,25].

Because *M. bovis* does not have a cell wall, it is intrinsically resistant to several antimicrobial classes, such as β-lactams. Several antimicrobials that are active against *M. bovis*, such as tetracyclines, macrolides, and fluoroquinolones, are also commonly used in antimicrobial metaphylaxis for the control of BRD, and there is mounting evidence that antimicrobial resistance among *M. bovis* is growing [26,27,28]. For example, one study that followed two cohorts of feedlot cattle documented that the resistance of *M. bovis* to enrofloxacin, florfenicol, clindamycin, and tulathromycin increased over an eight-year period [29].

Recent advancements in bacterial whole-genome sequencing (WGS) have allowed for the rapid comparison of genomic profiles across numerous organisms in relation to virulence and antimicrobial resistance, including *M. bovis* [30]. However, due to the difficulty of isolating and culturing *M. bovis*, there is a scarcity of information regarding the genomic determinants of virulence and antimicrobial resistance and their distribution. For example, as of December 2024, there are only 631 *M. bovis* genomes publicly available in the NCBI, a fourth of the more than 2400 publicly available for *Mannheimia haemolytica,* another bacterial BRD pathogen [31]. Therefore, the objective of our study was to use WGS to characterize the virulence and antimicrobial resistance genomic profiles of *M. bovis* recovered from lung lesions of deceased cattle submitted for necropsy to the Texas Veterinary Medical Diagnostic Laboratory.

## 2. Materials and Methods

### 2.1. Bacteriological Culture

Fifty-four isolates displaying *Mycoplasma*-like growth were recovered from bovine lung lesions by the Texas Veterinary Medical Diagnostic Laboratory (TVMDL, Canyon, TX, USA) in 2021 (n = 5) and 2022 (n = 49) and were transported to the Texas A&M Veterinary Education, Research, and Outreach (VERO) laboratory (Canyon, TX, USA). These isolates were initially inoculated in 5 mL of PleuroPneumonia-Like Organism (PPLO) broth (Remel, San Diego, CA, USA) and incubated at 37 °C with 5% CO_2_ for 5–7 days or until opalescent turbidity was observed [32]. Four 10 µL aliquots per tube were then spotted onto PPLO agar plates (Remel, San Diego, CA, USA) and incubated for 3–5 days under the same conditions. Colonies were then visualized under a Nikon SMZ 745T stereomicroscope (Nikon, Melville, NY, USA) and carefully picked from the agar plate using a 10 µL pipette tip and used to inoculate a 1.5 mL microcentrifuge tube containing 500 µL of PPLO broth. These tubes were then incubated for 24 h at 37 °C with 5% CO_2_ and used to inoculate a final 5 mL PPLO broth tube, which was incubated for 3–5 days at 37 °C with 5% CO_2_ prior to DNA extraction.

### 2.2. DNA Extraction and Quality Control

Each pure isolate in PPLO broth was centrifuged in two consecutive rounds, a 1 mL aliquot followed by a 500 µL aliquot, to pellet the cells prior to extraction. DNA was extracted using the DNEasy Ultraclean Microbial Kit (Qiagen, Hilden, Germany) on a QIAcube Connect RNA/DNA extraction instrument (Qiagen, Hilden, Germany) according to the manufacturer’s instructions. A heat treatment (65 °C for 10 min) was used for cell lysis instead of bead-beating to minimize DNA shearing, as recommended by the manufacturer’s instructions.

### 2.3. Mycoplasma bovis qPCR

To confirm that these isolates were *M. bovis*, a species-specific qPCR targeting the *uvrC* gene was performed. Briefly, a final 20 μL solution containing 1X Quantabio Perfecta SYBR FastMix low ROX buffer (Quantabio, Beverly, MA, USA), 500 nM of each primer [33], and 2 μL of DNA template was subjected to the following PCR program in a QuantStudio3 real-time PCR system (ThermoFisher Scientific, Waltham, MA, USA): a pre-cycling stage of 50 °C for 2 min and 95 °C for 10 min, followed by 40 cycles of 95 °C for 15 s, 60 °C for 30 s; and a melting curve stage of 95 °C for 15 s, 60 °C for 1 min, and a ramp to 95 °C (held for 1 s). Cycle threshold (Ct) values < 35 and a melting curve identical to that of the positive control (*M. bovis* ATCC 25523 strain) were required for identification of the isolates as *M. bovis*.

### 2.4. Whole Genome Amplification

Whole genome amplification was used to improve DNA yields. The REPLI-g Midi kit (Qiagen, Hilden, Germany) was utilized and concentrations were measured on a Qubit 4 fluorometer (ThermoFisher Scientific, Waltham, MA, USA) at a 1:100 dilution. To improve DNA quality, samples were debranched using T7 endonuclease (New England Biolabs, Ipswich, MA, USA) and custom bead cleaning was performed as described in the Ligation sequencing gDNA–whole genome amplification SQK-LSK112 protocol (Oxford Nanopore Technologies, Oxford, UK).

### 2.5. Nanopore Sequencing

Prior to DNA library preparation, samples were sheared to 8 kbp using g-TUBEs (Covaris, Woburn, MA, USA) with the manufacturer’s recommended settings (two centrifugations at 7200 rpm for one minute each time) in an Eppendorf 5424 centrifuge (Eppendorf, Hamburg, Germany). The sheared DNA (in 150 μL of low EDTA TE buffer) was cleaned with AMPure XP beads (Beckman Coulter, Brea, CA, USA) using a ratio of 0.4× and eluted into 14 μL of molecular-grade water (Corning, Corning, NY, USA). DNA concentrations were assessed using a NanoDrop Eight device (ThermoFisher Scientific, Waltham, MA, USA) and fragment sizes were assessed via visualization of the electropherogram in a genomic ScreenTape (Agilent, Santa Clara, CA, USA). DNA libraries were prepared using the native barcoding kit v14 (SQK-NBD114-24), and samples were sequenced in R10.4.1 flow cells in a MinION Mk1C sequencer (Oxford Nanopore Technologies, Oxford, UK) multiplexing 24 *M. bovis* isolates per flow cell. Two extraction blanks were included as negative controls to assess the presence of background DNA and the type strain ATCC 25523 (American Type Culture Collection, Manassas, VA, USA) was included as a positive control. Isolates that could not be successfully assembled and isolates with poor assembly quality (<100 coverage of the largest contigs or 10 or more contigs in the assembly) were reprocessed.

### 2.6. Bioinformatics and Statistical Analysis

All bioinformatics software was used with default parameters unless otherwise specified. Sequence quality was assessed using LongQC (version 1.2.0) [34], and de novo assemblies were derived using Flye (version 2.9.1) [35]. Assemblies were polished using Medaka (version 1.7.2) [36] and their assembly quality was assessed using QUAST (version 5.0.2) [37]. Assemblies were annotated using Prokka (version 1.14.5) [38], with the *M. bovis* PG45 genome available from NCBI (Accession ASM18338v1) as a reference. The presence of single-nucleotide polymorphisms (SNPs) in the core genome was inferred using Snippy (version 4.6.0) [39]. Roary (version 3.13.0) was used to estimate the pan-genome [40]. ABRicate (release FOFN MOTD 80 80) [41] was used to search the assemblies for virulence genes using the extended Virulence Factor Database [42] and for antimicrobial resistance genes using MEGARes (version 2.0) [43] and Resfinder [44]. The PlasmidFinder database was used by ABRicate to identify plasmid replicons [45]. Scoary was used to evaluate the associations between individual virulence genes and metadata points using Fisher’s exact test while controlling the false discovery rate with the Benjamini–Hochberg method according to a *p*-value cutoff of 0.2 [46].

### 2.7. Phylogenetic Analysis

The output of Snippy was used to create a maximum-likelihood phylogenetic tree in IQ-TREE (version 2.3.6) using the model finder with the ascertainment bias correction feature. Bootstrap estimates were obtained from 1000 ultrafast bootstrap iterations [47]. This tree was visualized in the interactive Tree of Life (iTOL version 7.0) [48].

## 3. Results

### 3.1. Description of Isolates

Out of 54 *M. bovis* isolates received from the TVMDL, 32 were successfully subcultured and confirmed to be *M. bovis* via species-specific qPCR. These isolates came from Texas (n = 13), New Mexico (n = 15), Oklahoma (n = 2), and Kansas (n = 1). These isolates originated from both dairy operations (n = 16) and beef operations (n = 11), as well as from both dairy breeds (n = 14) and beef breeds (n = 10). Isolates were retrieved from male (n = 11) and female (n = 5) calves. The calf age at collection was dichotomized into <100 days old (n = 14) and >100 days old (n = 11). Instances of missing data were present in multiple variables, as is often the case with diagnostic lab data, due to a lack of standardization regarding submitted sample information. There were *M. bovis* isolates lacking information for calf sex (n = 16), breed (n = 8), age (n = 7), type of operation (n = 5), or sample origin (n = 1).

### 3.2. Nanopore Sequencing and Bioinformatic Pipeline Performance

The *M. bovis* isolates had an average DNA concentration of 733 ng/μL (SD = 257) after whole genome amplification. The long reads maintained a median average Q score of twenty or greater for up to 20,000 bases. The average GC content of the *M. bovis* isolates was 29.3% of the genome. Eleven isolates were assembled into three contigs or less, while twenty-one isolates were assembled into more than three contigs. A majority (n = 25) of the *M. bovis* isolates contained at least one closed contig. The mean NG50 and LG50 were 793,671.14 nucleotides and 1.25 contigs, respectively.

### 3.3. Pangenome Analysis

**Figure 1:**
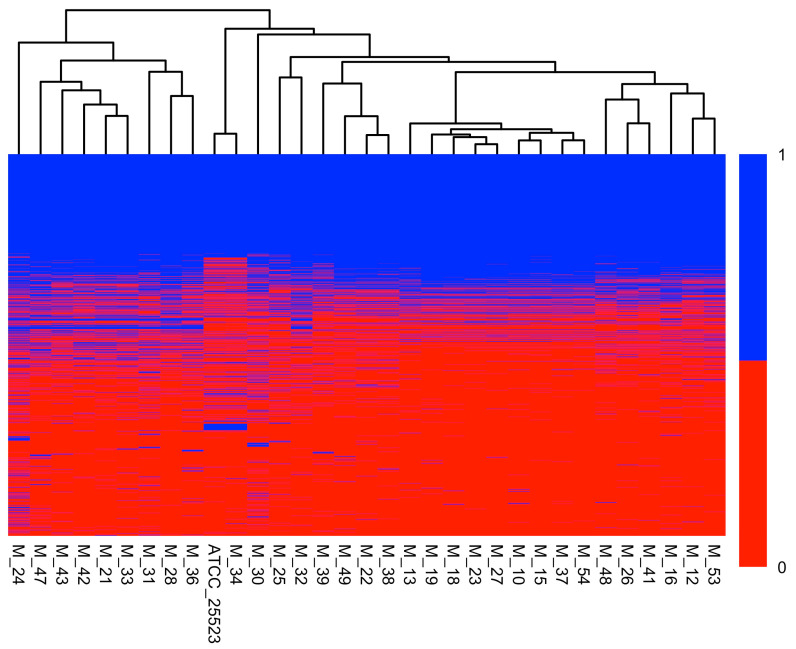
Heatmap depicting the distribution of all genes among the *Mycoplasma bovis* isolates. Genes are color-coded according to their presence (blue) or absence (red). The dendrogram represents hierarchical clustering with the complete linkage method and Jaccard distances.

Roary identified a total of 2054 genes in the pangenome of the *M. bovis* isolates. Of these genes, 535 were classified as core genes (present in 99–100% of the genomes), 22 were classified as soft core genes (present in 95–99% of the genomes), 587 were classified as shell genes (present in 15–95% of the genomes), and 910 were classified as cloud genes (present in <15% of the genomes). A heatmap of the pangenome is shown in Figure 1.

### 3.4. Virulence and Antimicrobial Resistance Genes

**Figure 2:**
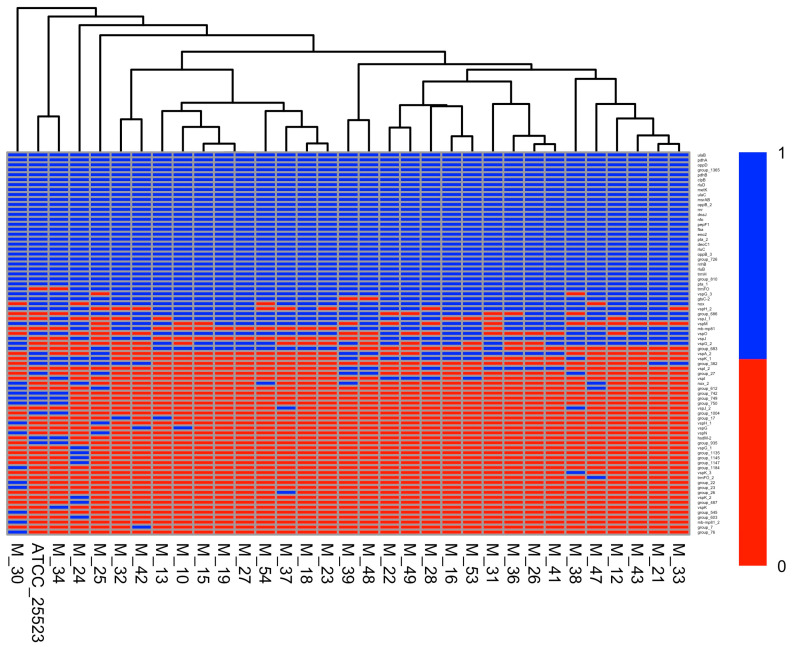
Heatmap displaying the distribution of virulence genes in the *Mycoplasma bovis* isolates. Genes are color-coded according to their presence (blue) or absence (red). The dendrogram represents hierarchical clustering with the complete linkage method and Jaccard distances.

Using ABRicate in conjunction with the extended virulence factor database only identified three virulence genes: *tuf* elongation factor Tu, *pdhB* alpha-ketoacid dehydrogenase subunit beta, and *p48* BMC family ABC transporter substrate-binding protein. These genes were present in all isolates and, because of this, Scoary was unable to identify any associations between the genes and the available metadata variables. This led us to create a manually curated list of previously described virulence genes [20,21,22,23,24,25,49] from the output of Roary. We identified an additional 76 virulence-associated genes present in the *M. bovis* isolates. Twenty-eight of these genes were present in all isolates and can therefore be considered part of the core genome according to the cutoff values utilized by Roary. One gene, *trmFO*, present in all but one isolate, can be considered a soft core gene. The remaining genes can be placed either in the shell genome (n = 15) or in the cloud genome (n = 32). A summary of these genes can be found in Table S1. A heat map showing the distribution of virulence genes among the *M. bovis* isolates is shown in Figure 2.

ABRicate did not find any antimicrobial resistance genes using MEGARes or ResFinder. No additional antimicrobial resistance genes were identified while manually curating the output from Roary. No plasmids could be identified via ABRicate while using the PlasmidFinder database.

### 3.5. Statistical Analysis with Scoary

We first ran Scoary on the unmodified output from Roary and it was unable to identify any statistically significant associations between any genes and metadata variables. Running Scoary on the manually curated virulence gene dataset, however, yielded several statistically significant associations, which are summarized in Table 1. Notably, several genes encoding for variable surface proteins were more prevalent in isolates from beef cattle and beef operations.

### 3.6. Phylogenetic Analysis

**Figure 3:**
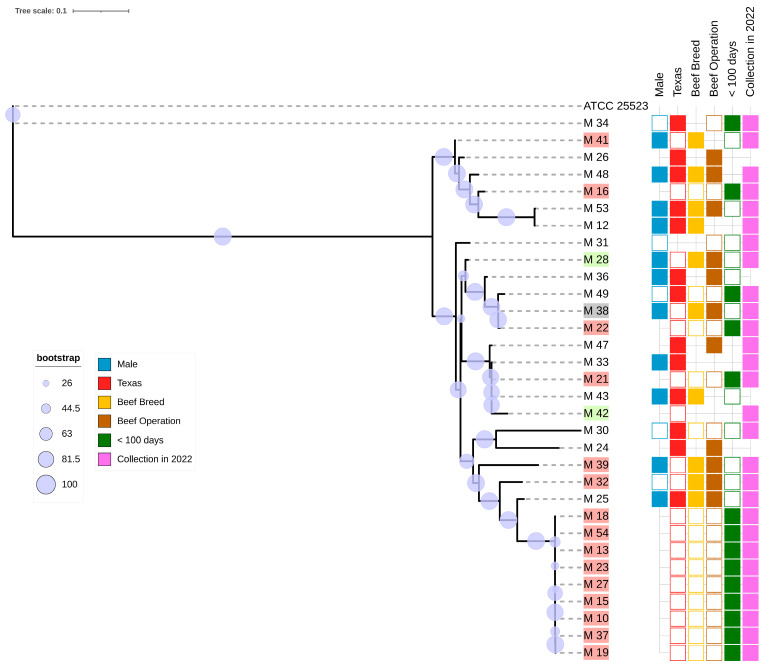
Core SNP-based maximum-likelihood phylogenetic tree. The bootstrap values are represented by circles of different sizes. The scale bar represents the average nucleotide substitution per SNP in the phylogeny tree. The table summarizes the available metadata (color-coded by variable) per isolate. The male variable was dichotomized into male (full square) and female (empty square). The Texas variable was dichotomized into origin in Texas (full square) and outside of Texas (empty square). The names of the isolates from outside the state of Texas are highlighted and color-coded by state of origin: New Mexico is highlighted in red, Oklahoma is highlighted in green, and Kansas is highlighted in gray. The beef breed variable was dichotomized into beef breed (full square) and dairy breed (empty square). The beef operation variable was dichotomized into beef operation (full square) and dairy operation (empty square). The <100 days variable was dichotomized into age < 100 days (full square) and age > 100 days (empty square). The collection in 2022 variable was dichotomized into samples collected in 2022 (full square) and those collected in 2021 (empty square). No square indicates missing data.

The best nucleotide substitution model selected by IQ-TREE was the transversion model with an unequal base frequency. The core SNP-based phylogenetic tree constructed in IQ-TREE and visualized iTOL is shown in Figure 3. Clustering based on animal sex, state of origin, breed, and type of operation can be observed. Of note is the clade consisting of the isolates M_18, M_54, M_13, M_23, M_27, M_15, M_10, M_37, and M_19. In addition to clustering together, the metadata for these isolates are identical across variables: each *M. bovis* isolate was recovered from a lung specimen of a dairy animal of unknown sex from New Mexico, and the collection of the specimens was on the same date.

## 4. Discussion

The purpose of this study was to describe the genomic features associated with the virulence and antimicrobial resistance of *M. bovis* isolated from cattle in the Texas panhandle. The results of our phylogenetic and pangenomic analysis showed a great deal of genetic diversity among the *M. bovis* circulating among cattle in the Texas panhandle. This diversity was also reflected in the virulence profiles of the isolates. No antimicrobial resistance genes were detected among the isolates in this study.

Clustering based on animal age, sex, and breed, state of origin, and the type of operation can be observed among the *M. bovis* isolates in this study. Interestingly, one of our isolates, M_34, appears to be very similar to the ATCC strain used in this study as a reference. This isolate came from a dairy calf in Texas that was less than 100 days old. Because our phylogenetic analysis utilized the SNPs present in the core genome, diversity in the accessory genome was not captured with this technique. Examining the pangenome and virulence profiles of these strains reveals key genomic differences. For example, the *vspK* gene appears in M_34 but not ATCC_25523. A similar phenomenon can be observed within the clade composed entirely of dairy calves less than 100 days old from New Mexico, with all the samples of this clade collected and received on the same date. Because of the nature of our metadata, there is no way to definitively determine that these isolates originated from the same dairy farm, though this hypothesis seems plausible given the identical metadata for these isolates. Similarly to the relationship between M_34 and ATCC 255253, our core SNP phylogenetic tree may not adequately reflect all the genomic differences between the isolates in this clade. Indeed, key differences emerge when examining the *vsp* genes present in these isolates. For example, three isolates in this clade, M_10, M_13, and M_37, possess unique *vsp* genes: *vspG*, *vspJ*, and *vspK*, respectively.

Our results also show that a significant proportion of the virulence genes of any given isolate are present in the core genome, as indicated by their ubiquitous distribution among our isolates. Among these are several multifunctional enzymes with both a metabolic and virulence-associated role. One of these is the *tuf* gene coding for the Elongation factor Tu (EF-Tu), a protein that can be found in many bacterial genera, and its primary function is to catalyze the binding of aminoacyl t-RNA to ribosomes, enabling translation [50]. However, this protein has a wide array of other known functions, such as the modulation of the host immune response, adhesion to host epithelial tissue, and the regulation of cell shape [50]. Though the role of EF-Tu in *M. bovis* infection remains poorly understood, previous work has shown that EF-Tu in *Mycoplasma hyopneumoniae* is critical to its attachment to tracheal epithelial cells and evasion of the host immune system in swine [51,52]. One *M. bovis* inoculation study showed that naïve bison immunized with an injectable subunit vaccine containing EF-Tu displayed reduced joint infection, lung lesions, and lung bacterial loads [53]. Given the uniform distribution of this gene among our isolates, and the previously characterized role EF-Tu has in cytoadherence, EF-Tu may also be a viable vaccine candidate in cattle.

Other virulence factors were present in most, but not all of our isolates, such as the shell gene, *nox*, which was present in 88% of the isolates. This gene codes for an oxidoreductase enzyme with dual roles in the production of H_2_O_2_ and cytoadherence, which may contribute to the formation of caseonecrotic lesions [18].

The genes coding for membrane lipoprotein p81 (*mb-mp81*) and a glycerol ABC transporter permease (*gtsC-2*) were also present in the shell genome at 59% and 94% prevalences, respectively. Both genes are associated with the survival and proliferation of *M. bovis* in bovine kidney cells [20]. Though the roles of these genes in its pathogenesis remain largely unknown, their presence in the shell genome suggests they are a dispensable component of the virulence of *M. bovis*.

The pangenome analysis identified a total of twenty-two genes coding for variable surface lipoproteins, including *vdpK*, *vspO*, *vspN*, *vspH*, *vspI*, *vspG*, and *vspM*. These results highlight the previously described role of variable surface lipoproteins in generating antigenic diversity [54]. The statistically significant associations between metadata variables and these specific Vsps suggest that the distribution of these genes may be associated with host factors such as animal age, animal breed, and the type of operation.

The tools used in this study were unable to identify any antimicrobial resistance genes in the *M. bovis* isolates. Previous WGS-based analyses have identified mutations in multiple genes, such as *gyrA* and *parC,* that confer resistance to antimicrobials [55,56]. Our results may highlight a limitation of using computational tools such as ABRicate to identify specific antimicrobial resistance genes present in databases for *M. bovis* rather than mutations associated with drug resistance.

Though this study increases our understanding of the genetic diversity of *M. bovis*, there are some notable limitations. These isolates were derived from the convenience sampling of animals submitted to the TVMDL for necropsy. Because of this, our results may be influenced by selection bias, given that no isolates from healthy cattle were included. Additionally, the nature of convenience sampling, combined with our small sample size, makes it difficult to generalize our findings to the wider population of Texas panhandle cattle. Many samples also included instances of missing metadata, as the level of detail provided in the sample metadata is at the whim of the client submitting the sample. These limitations may make it difficult to ascertain the biological significance of the statistical associations between the *M. bovis* genes and the available metadata in this study. However, the trends in virulence gene carriage presented here should be further explored. Further studies should include a larger sample size, the sampling of healthy cattle, and standardized metadata collection to better capture the diversity of *M. bovis* in the Texas panhandle and the biological significance of the statistical associations presented here.

## 5. Conclusions

This study provides greater insight into the genetic diversity of *M. bovis* isolates circulating among cattle around the Texas Panhandle in the USA. Additionally, our results showed a heterogeneous distribution of virulence determinants among these isolates. Further research is needed to fully understand the role of these virulence determinants in the pathogenesis of *M. bovis* infections in cattle.

## Figures and Tables

**Table 1:** The association of *Mycoplasma bovis* virulence genes with the characteristics of their cattle hosts.

Gene	Beef Breed	Beef Operation	<100 Days Old
*mb-mp81*	OR = 22.5 Adj *p*-value = 0.081	OR = 30 Adj *p*-value = 0.066	
*vspO*	OR = 14.3 Adj *p*-value = 0.093	OR = 0.07 Adj *p*-value = 0.180	OR = 34.7 Adj *p*-value = 0.088
*vspA*	OR = 19.5 Adj *p*-value = 0.093	OR = 12.3 Adj *p*-value = 0.180	OR = 0.04 Adj *p*-value = 0.120
*vspI*	OR = Inf Adj *p*-value = 0.119		OR = 0 Adj *p*-value = 0.119
*vspJ*			OR = 0.09 Adj *p*-value = 0.158
*vspI*	OR = Inf Adj *p*-value = 0.119	OR = Inf Adj *p*-value = 0.184	

Beef breed, beef operation, and age <100 days old were used as the variables under analysis, with dairy breed, dairy operation, and age >100 days old as the reference groups. The odds ratios (ORs) were obtained via Scoary. OR values of Inf (infinity) or 0 indicate complete separation of the data. Adj *p*-value: the false discovery rate was controlled using the Benjamini–Hochberg method and according to a *p*-value cutoff of 0.2.

## Data Availability

De-identified datasets from this study are available in the SRA archives at the NCBI (https://www.ncbi.nlm.nih.gov/sra/PRJNA1194628, accessed on 4 February 2025).

## Supplementary Materials

The following supporting information can be downloaded at https://www.mdpi.com/article/10.3390/pathogens14020167/s1, Table S1: virulence-associated genes present in the *Mycoplasma bovis* isolates.
